# Cyclic voltammetry as a measuring tool in Parkinson’s disease and associated psychiatric commorbidities

**DOI:** 10.1192/j.eurpsy.2023.1597

**Published:** 2023-07-19

**Authors:** C. A. Ciobanu, I. Ionita, A.-I. Mihailescu, A.-M. Ciobanu

**Affiliations:** 1 Faculty of Medicine, “Titu Maiorescu” University, Bucharest; 2 Faculty of Medicine, “Carol Davila” University of Medicine and Pharmacy; 3 Ist Department, “Prof. Dr. Alex Obregia” Hospital, Bucharest, Romania

## Abstract

**Introduction:**

Despite the rapid increase in disability and death due to Parkinson’s disease and associated psychiatric comorbidities (psychosis, depression, cognitive impairment, anxiety), the quest for a clear pathophysiological mechanism and treatment remains elusive. Numerous studies aim to identify a metabolomic fingerprint for PD and new, promising biomarkers are discovered with implications beyond neurodegenerative diseases, such as novel markers as predictors of bipolar type in depressed patients.Changes in neuronal microenvironment employ electrochemical techniques, such as cyclic voltammetry, used in both animal and human models of PD to monitor dopamine (DA) alterations in vivo, with high spatial and temporal resolution.

**Objectives:**

Our aim is to investigate the latest scientific literature on PD and associated neuropsychiatric disorders and review the applications cyclic voltammetry has in recent technological advances in the field.

**Methods:**

To gain a broad understanding of the subject, we have consulted multiple scientific literature databases (PubMed, Google Academic, Science Direct) using the keywords “cyclic voltammetry, Parkinson’s disease, psychiatric disorders, dopamine” and included original research articles published in the last 10 years

**Results:**

The first *in situ* measurement of DA release in the human brain has been demonstrated in a sequential investment task, with implications for future research in decision-making behavior.

One study combines cyclic voltammetry and wireless telemetry for in vivo recording of changes in extracellular levels of DA, with high temporal and spatial resolution.

Disulfide nanorod-graphene-β-cyclodextrin nanocomposites biosensors have been succesfully used in detecting DA in rodent brain and human blood serum samples, with implications for minimally invasive measuring techniques.

Animal studies use cyclic voltammetry to monitor changes in DA levels in cerebrospinal fluid and plasma of mouse models of PD and investigate DA metabolism, release, uptake and receptor sensitivity in Knock-out mice, with implications for the diurnal variation of extracellular DA tone and release.Furthermore, a human alpha-synuclein-expressing mouse model of PD exhibited increased extracellular DA concentrations, decreased DA uptake and relieved paired-stimulus depression.

**Conclusions:**

Cyclic voltammetry is a powerful tool in the expansion to humans of electrochemical recording techniques in PD. The final aim is to investigate DA neuron physiology before neurodegeneration onset and to measure neurotransmitter release in real time.

**Disclosure of Interest:**

None Declared

